# Refined Techniques for Enabling Long-Term Cryo-Repository Using Vitrification and Laser Warming

**DOI:** 10.3390/bioengineering10050605

**Published:** 2023-05-18

**Authors:** Chiahsin Lin, Wen-Chung Hsieh, Kanokpron Loeslakwiboon, Cheng-Liang Huang, Ting-Chun Chen, Sujune Tsai

**Affiliations:** 1 National Museum of Marine Biology & Aquarium, Pingtung 94450, Taiwan; 2 Institute of Marine Biology, National Dong Hwa University, Pingtung 94401, Taiwan; 3 He Wei Precision Company Limited, Hsinchu 30271, Taiwan; 4 Department of Applied Chemistry, National Chiayi University, Chiayi 600355, Taiwan; 5 Department of Post-Modern Agriculture, Mingdao University, Chang Hua 52345, Taiwan

**Keywords:** cryobanking, cryo-jig, cryopreservation

## Abstract

Vitrification and ultrarapid laser warming are crucial for the cryopreservation of animal embryos, oocytes, and other cells of medicinal, genetic, and agricultural value. In the present study, we focused on alignment and bonding techniques for a special cryojig that combines a jig tool and jig holder into one piece. This novel cryojig was used to obtain a high laser accuracy of 95% and a successful rewarming rate of 62%. The experimental results indicated that our refined device improved laser accuracy in the warming process after long-term cryo-storage through vitrification. We anticipate that our findings will lead to cryobanking applications that use vitrification and laser nanowarming to preserve cells and tissues from a wide range of species.

## 1. Introduction

Cryopreservation is widely used in long-term repositories despite requiring the freeze–thaw process, which can severely affect gamete function. Cryopreservation techniques require balancing the freezing rate with the need to regulate cellular dehydration and minimize intracellular ice formation [1]. Over the past few decades, cryopreservation has become a well-established approach for preserving human [2], cow [3], and mouse [4] cells and tissues. The cryopreservation of mammalian embryos and oocytes can be conducted through slow cooling or vitrification. Although these two cryopreservation methods differ considerably, both require optimal conditions, which are controlled during each stage of the procedure. In addition, the cryopreservation of gametes, embryos, and embryonic cells has brought tremendous value to aquatic biotechnology and become a successful approach for propagating economically important species, protecting endangered species, and maintaining genetic diversity [5]. To date, the spermatozoa of hundreds of fish species have been cryopreserved [6]; however, the cryopreservation of fish oocytes and embryos remains challenging, and related research remains in the initial stages.

Vitrification is a safe and promising alternative to conventional slow-rate freezing [7,8,9]. Vitrification is used to cool vertebrate and invertebrate biomaterial to avoid ice crystal formation and utilizes high concentrations of cryoprotectant and rapid cooling rates to directly transform a solution into a clear, noncrystalline, solid-state compound that resembles glass [10,11]. Recently, another encouraging method in the cryopreservation of vertebrate germplasm—laser warming with gold nanoparticles (GNPs)—has been developed [12,13], and it has been used to effectively reanimate vitrified zebrafish embryos [14] and mouse oocytes and embryos [4,15]. Laser nanowarming can aid in the rescue of cryoprotectant-sensitive coral larvae and can provide rapid and uniform warming rates to minimize damage to coral larvae by ice crystallization [16]. Related achievements include the first successful cryopreservation of *Seriatopora caliendrum* and *Pocillopora verrucosa* larvae with the presence of Symbiodiniaceae and the vertical transmission of algal symbionts, which were able to settle after vitrification and laser warming [17,18]. Long-term cryo-repositories after vitrification and subsequent laser warming might be achievable using a device we developed in our previous study [19]; however, this device requires two jigs and fitting positions as well as cryostick assembly procedures to achieve high laser accuracy and effective rewarming. The future of long-term cryobanking requires improved efficiency and sustainable, innovative solutions.

Recently, our laboratory employed two cryojigs to load and warm samples for vitrification and laser warming; using these cryojigs, we obtained a laser accuracy of 76% [19]. This low accuracy may have been caused by the laser machine and jig axis circle being imperfectly aligned and not centered on the origin. The goal of the present research was to optimize the mechanical arrangement of our device and cryojigs to enable the laser to hit a designated target after long-term cryo-storage with greater accuracy and achieve successful laser warming without recrystallization.

## 2. Materials and Methods

### 2.1. Alignment of Customized Device and Laser Machine

**Figure 1:**
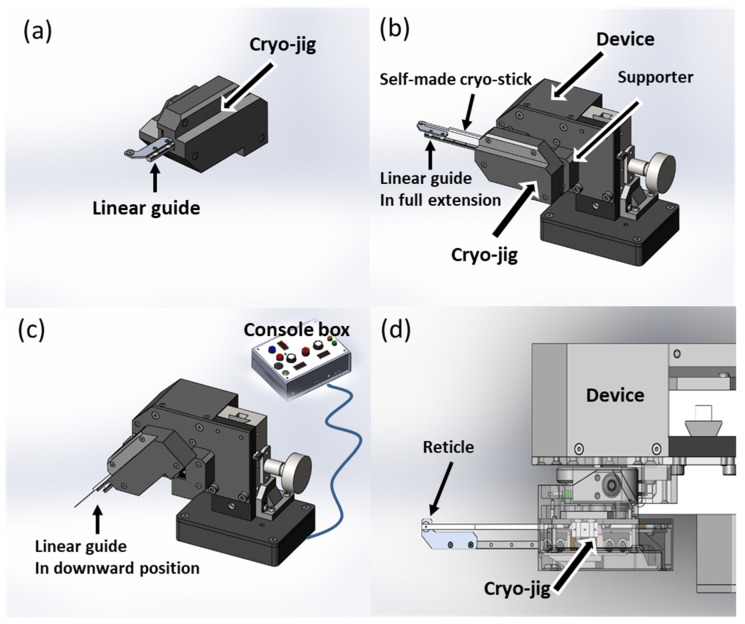
Alignment of laser machine with device and cryojig. (**a**) Structure of cryojig with linear guide. (**b**) The cryojig was placed on the supporter of the device with the cryostick and linear guide in full extension. At this time, the device could be turned on and moved to an appropriate position underneath the laser machine. (**c**) The jig was swung into the downward position, which (**d**) activated the machine, which fired the laser when the cryojig swung back to the upward position. On the basis of the deviation between the position where the laser hit and the targeted position, the position of the device was repeatedly adjusted until the laser precisely hit the target.

**Figure 2:**
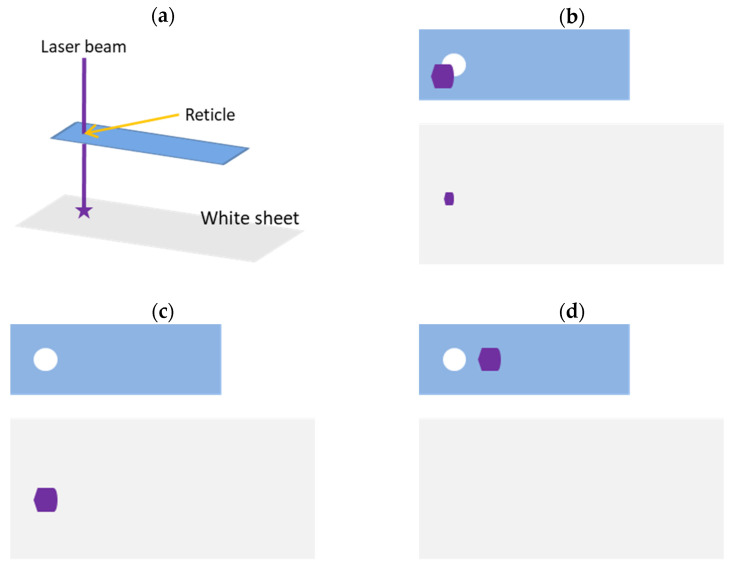
Three possible configurations of the cryojig relative to the laser. (**a**) The alignment of the reticle and the laser. The white sheet was used to indicate where heat from the laser beam led to carbonization. (**b**) State 1: When the laser beam hit the reticle margin, the beam penetrated only partially. (**c**) State 2: Full penetration of the laser beam. (**d**) State 3: The laser beam hitting the linear guide.

The precise alignment of a laser machine is essential for the laser to effectively hit a sample (Figure 1). The laser machine employed in this study (iWeld 980 Series, 60 J, Nd:YAG infrared laser, LaserStar Technologies Corporation, Riverside, CA, USA) was equipped with a stereomicroscope with an ocular reticle, which facilitated sample observation and laser alignment. Our original cryojig was used to lower a sample into liquid nitrogen for vitrification and long-term cryo-storage, raise the sample into the laser focal region, and trigger the laser for warming. The modified version of our developed cryojig allowed the cryostick with the blade to be easily replaced after each laser pulse to ensure consistent and reproducible sample warming. For testing, the laser was fired at the target site and passed through the center of the reticle with minimal touching of the reticle margin to determine the position of the cryojig relative to the laser machine (Figure 2). A 0.8-μL droplet of vitrification solution (VS consisting of 2 M EG, 1 M PG, 40% *w*/*v* Ficoll, and 10% *v*/*v* GNPs at a final concentration of 1.2 × 10^18^/m^3^) was placed on the custom cryostick, which was centered in the reticle of the cryojig. The cryojig was secured in a designated position, and another laser was emitted to ensure precise alignment to hit the droplet. The energy provided by a single laser pulse could be varied through modulation of the input voltage and pulse duration; a laser calibration system based on a laser power meter was created to determine the amount of energy in each laser pulse. The alignment process was repeated every 10 or fewer pulses to ensure that the laser footprint fully covered the sample. 

### 2.2. Cryopreservation

**Figure 3:**
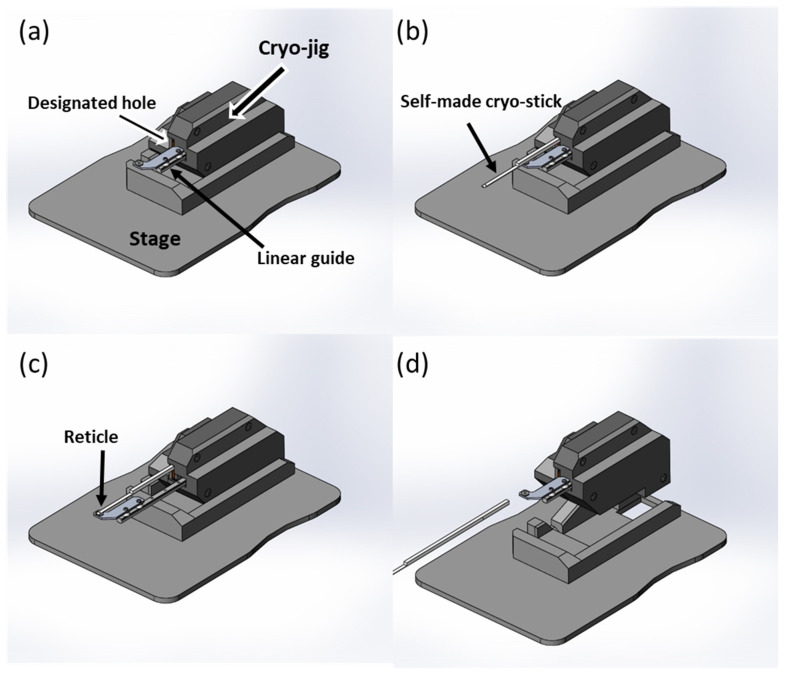
Cryopreservation procedure. (**a**) Customized cryojig and its stage. The designated hole and linear guide are visible on the cryojig. (**b**) The developed cryostick was inserted until it reached the stopper in the designated hole of the cryojig. (**c**) The linear guide of the jig was pulled out to full extension. The reticle of the linear guide was fixed at a relative position on the jig, and then a droplet of approximately 0.8 μL of VS was placed onto a cryostick blade directly above the reticle. (**d**) The cryostick was removed from the cryojig for cryopreservation in liquid nitrogen.

Our cryojig and cryostick were used to rapidly cool VS droplets by plunging them into liquid nitrogen for long-term cryo-storage and to allow equilibration to the temperature of the liquid nitrogen (Figure 3). The developed cryostick was inserted into the designated hole of the cryojig until it reached the cryojig stopper. The cryojig’s linear guide was fully extended while the mechanism of the cryojig remained still. The reticle of the linear guide was aimed at a location on the cryojig, and a VS droplet was placed on the cryostick blade directly above the reticle. The cryostick was promptly immersed in the liquid nitrogen after being removed from the cryojig.

### 2.3. Testing of Laser Accuracy and Successful Rewarming Rate

**Figure 4:**
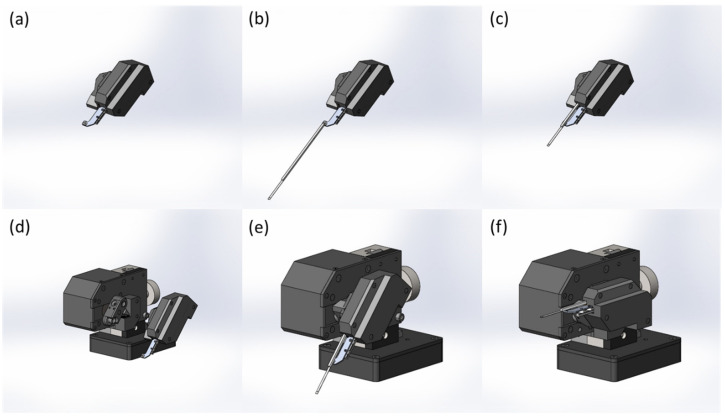
Laser warming procedure. (**a**) The cryojig was positioned for laser warming. (**b**) The sample on the blade of the cryostick was maintained in liquid nitrogen, while the rest of the cryostick was not. (**c**) The main stick component of the cryostick was then placed into the designated hole of the cryojig until the deepest stopper was reached. (**d**,**e**) The cryojig was mounted onto the supporting arm of the device, which had a mechanism that automatically fixed the cryojig in the correct position, oriented downward at a 45° angle. (**f**) The device swung the cryojig with the cryostick from the downward to the upward position into the laser focal region after the actuator was engaged. When the cryostick reached the upward position, the laser pulse was instantly activated for warming.

A 1-ms high-energy laser pulse was aimed at the VS droplets, and the laser accuracy and successful rewarming rate were measured. Before laser warming, the cryojig was attached to the laser machine, which was constructed from conventional bioplastic polylactic acid and an ionic polymer–metal composite (Figure 4). When the cryostick was retrieved after being frozen, the sample on the blade remained in the liquid nitrogen, and the main stick component was exposed to the open air; the stick component was then inserted into the designated hole of the cryojig until it reached the deepest stopper. The cryojig was then mounted in a downward orientation at approximately 45° on the supporting arm of the device, which had a mechanism designed to hold the cryojig in place. After the digitized actuator was activated, the device swung the cryojig and cryostick into an upward position, raising the sample into the laser beam focal region; this motion was completed using three different tip travel speeds (1113 mm/s, 834 mm/s, and 668 mm/s; 60 replicates for each of the 3 conditions), the results of which are compared in the Results and Discussion sections. By contrast, the downward position allowed the sample to be placed directly into the liquid nitrogen for vitrification. Attached control knobs were used to adjust the rotary movement of the cryojig. The digitized actuator moved the central platform supporting the cryojig; the movement parameters for the cryojig and sample were adjusted with the control knobs, ensuring the platform moved at a suitable velocity and stopped in the desired position. The robotic arm system of the cryojig rotated from the downward to the upward position when the actuator was engaged. To minimize the risk of accidents, the central console and laser device each included two safety buttons that governed the activation of the laser pulse. The visual observation was conducted to determine whether any ice formed inside the sample.

**Figure 5:**
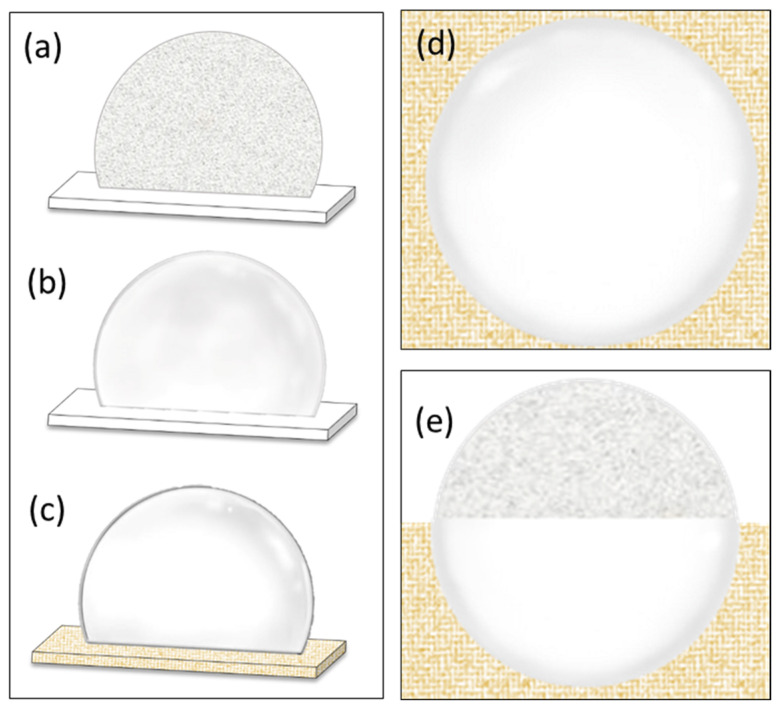
Observations of devitrified and vitrified droplets. (**a**) A devitrified droplet of VS demonstrating ice crystal formation. (**b**) A translucent vitrified droplet prior to laser warming. (**c**) The crystal-clear vitrified droplet after laser warming. The yellow area depicts where the laser was projected. (**d**) A vitrified droplet after exposure to the full diameter of the laser beam and (**e**) a devitrified droplet partially exposed to the beam.

The laser accuracy and successful rewarming rates were determined using the following principles (Figure 5). When the laser was fired, lasers that hit and missed the target as well as partial and complete hits of the target were respectively differentiated as follows. (1) A successful and complete laser hit turned the droplet transparent, but a miss turned the droplet from translucent to opaque and left a light-yellow mark at the point of contact. (2) A partial laser hit turned the point of contact transparent, while the other areas of the droplet became opaque; this opacity eventually spread to the rest of the droplet through crystallization. The successful rewarming rate was determined after only a complete hit of the droplet.

### 2.4. Statistical Analysis

The laser accuracy and successful rewarming rate achieved with different cryojig tip travel speeds were compared using SPSS version 17.0 (SPSS, Chicago, IL, USA). The data were examined for normality through a one-sample Kolmogorov–Smirnov test prior to an analysis of variance (ANOVA). Levene’s test was used to determine whether the variance was homogeneous (*p* > 0.05). Differences were determined by one-way ANOVA with Tukey post hoc tests. *p* < 0.05 indicated statistical significance.

## 3. Results and Discussion

The novel cryojig and device designed in this study could manipulate our custom cryostick during rapid cooling in liquid nitrogen and ultrarapid warming under laser pulses. Samples were cooled with a vitrification rate of 100% and rapidly warmed by laser pulses with an accuracy exceeding 90%; furthermore, these samples were successfully rewarmed at a maximum rate of 62% (Table 1). This work built upon our previous work [19], which described how a discretional jig tool (cryojig A) and jig holder (cryojig B) were aligned to enable a laser to hit the same target; however, in this study, we focused on developing alignment and bonding techniques to combine the jig tool and jig holder developed from the previous study into one piece to achieve a higher laser accuracy and a higher successful rewarming rate. The major function of the customized cryojig was to maintain the sample securely in place throughout laser operation to enable the adjustment of the process to increase the laser accuracy and vitrification rate and ensure successful laser rewarming. This laser accuracy (>90%) and successful rewarming rate (62%) achieved were significantly higher than the accuracy (76%) and rewarming rate (59%) achieved with the cryojigs in our previous study [19]. Greater accuracy results in more successful rewarming. The lower accuracy from our previous study might be attributed to the laser machine and jig axis circle being inadequately aligned and not centered on the origin.

In this study, the average laser energy was the mean energy per pulse for three tip travel speed settings that resulted in the rewarming of the droplet without any ice crystallization (i.e., the transition from glassy to cloudy). The droplets were warmed at the highest jig tip travel speed (1113 mm/s), and the laser accuracy and glassy droplet rate after laser rewarming were 95% and 60%, respectively. By contrast, when the droplets were warmed with a slower tip travel speed (668 mm/s), these rates were 92% and 55%. Although the difference in the results obtained at these two speeds was not statistically significant, the slower warming speed (longer exposure time of the sample in the air before the laser hit) was consistent with the lethality possibly ascribed to ice recrystallization during warming. Our lab continues to study the effects of higher speeds (i.e., ≥2000 mm/s) on the successful rewarming rate. Through such innovation, we can routinely position and align bonded components more efficiently and achieve high laser accuracy and successful rewarming of samples vitrified for cryopreservation. 

Various vitrification carriers, such as Cryotop, Cryoloop, and Cryohook, are used to manipulate samples during cryopreservation. One study suggested a device comprising a closed loop or an open hook made of plastic or metal wire that was attached to the end of a stem and used to collect biological samples [20]. The cryopreservation of animal cells or tissues was performed using a Cryotop device for vitrification [21]. Our developed cryostick measured 20 × 0.4 × 0.1 mm^3^ and consisted of a fine blade of transparent film attached to an acrylonitrile butadiene styrene (C_8_H_8_.C_8_H_6_.C_6_H_6_N)_N_ stick backing. Our device was specifically engineered for use with our one-piece cryojig. Approximately 62% of the samples vitrified with our custom cryostick were successfully rewarmed and remained in a vitrified state without ice crystal formation. The results of the present study strongly suggest that our cryostick coupled with a customized jig and device offers ultrarapid cooling as well as laser warming with minimal ice crystal formation. 

Vitrification prevents the formation of ice crystals inside cells by using cryoprotectants and rapid cooling, which enable cells to directly transition from a frozen state into a glassy and vitrified state without crystallization [4,15,22]. Although vitrification could be achieved with a lower cryoprotectant concentration and decrease the toxic effects on cells, this would increase the number of ice nuclei formed during the freezing process; rapid warming is essential to prevent intracellular ice formation [11]. Infrared laser warming can heat a sample droplet at up to 10^7^ °C/min, depending on the energy of the laser pulses delivered to the sample and the laser absorption capacity of the particles present [4]. The Figures illustrate a representative run of the system, in which a 0.8-µL aqueous sample (VS consisting of 2 M EG, 1 M PG, 40% *w*/*v* Ficoll, and 10% *v*/*v* GNPs at a final concentration of 1.2 × 10^18^/m^3^) was placed on the 0.1-mm blade of our developed cryostick; the cryostick was placed in our custom cryojig device, and the blade was cooled through immersion in liquid nitrogen. Subsequent laser warming was performed after the cryostick blade was raised out of the liquid nitrogen and positioned under the laser. The laser was fired for 0.25 s with a characteristic wavelength of 535 nm. For our purposes, once we empirically calculated the melting energy (voltage) for a particular droplet size, composition, and GNP concentration, we could consistently warm droplet preparations, illustrating one of the advantages of our device. We achieved a high rewarming rate of 62%. The concentration of GNPs (1.2 × 10^18^/m^3^) and laser settings (300 V, 10-ms pulse) used in the present study were selected to minimize the energy required to consistently warm 0.8-µL droplets while avoiding overheating and preventing ice recrystallization.

## 4. Conclusions

This research developed a method of routinely achieving the precise alignment of robust and ultrastable optical assemblies to achieve high performance and reliability in the long-term cryopreservation of biomaterials. We anticipate that as our approach is perfected, it will allow for further applications of cryobanking through the vitrification and laser nanowarming of a range of cells and tissues from diverse species.

## Figures and Tables

**Table 1:** Results obtained using cryojig and various tip travel speeds, including the average laser accuracy and successful rewarming rates (mean percentage values ± standard deviation) for cryopreservation.

	Percentage (%)		
Tip travel speed	1113 mm/s	834 mm/s	668 mm/s
Laser striking accuracy	95.00 ± 0 ^#^	93.33 ± 2.89 ^#^	91.67 ± 2.89 ^#^
Successful rewarming rate	60.00 ± 5.00 *	61.67 ± 2.89 *	55.00 ± 0 *

n = 60 for each of three conditions (N = 180 total). ^#^ indicates no significant differences in laser striking accuracy between the three speeds (*p* > 0.05). * indicates no significant differences in successful rewarming rate between the three speeds (*p* > 0.05).

## Data Availability

The data presented in this study are available on request from the corresponding author.
